# Commentary: Targeting the MRI-mapped psychopathology of major psychiatric disorders with neurostimulation

**DOI:** 10.3389/fpsyt.2022.990512

**Published:** 2022-09-20

**Authors:** Jia-Xin Xie, Jin-Jin Cui, Yang Cao, Yue-Wen Gu, Jing-Wen Fan, Lei Ren, Xiao-Fan Liu, Shu-Wan Zhao, Wang-Hong Shi, Qun Yang, Yin-Chuan Jin, Feng-Zhan Li, Lei Song, Hong Yin, Feng Cao, Baojuan Li, Long-Biao Cui

**Affiliations:** ^1^Department of Clinical Psychology, Fourth Military Medical University, Xi'an, China; ^2^The Second Medical Center, Chinese PLA General Hospital, Beijing, China; ^3^Department of Radiology, Xi'an People's Hospital (Xi'an Fourth Hospital), Xi'an, China; ^4^School of Biomedical Engineering, Fourth Military Medical University, Xi'an, China

**Keywords:** MRI, neurostimulation, psychopathology, localization, brain phenotype

## Introduction

**Figure 1:**
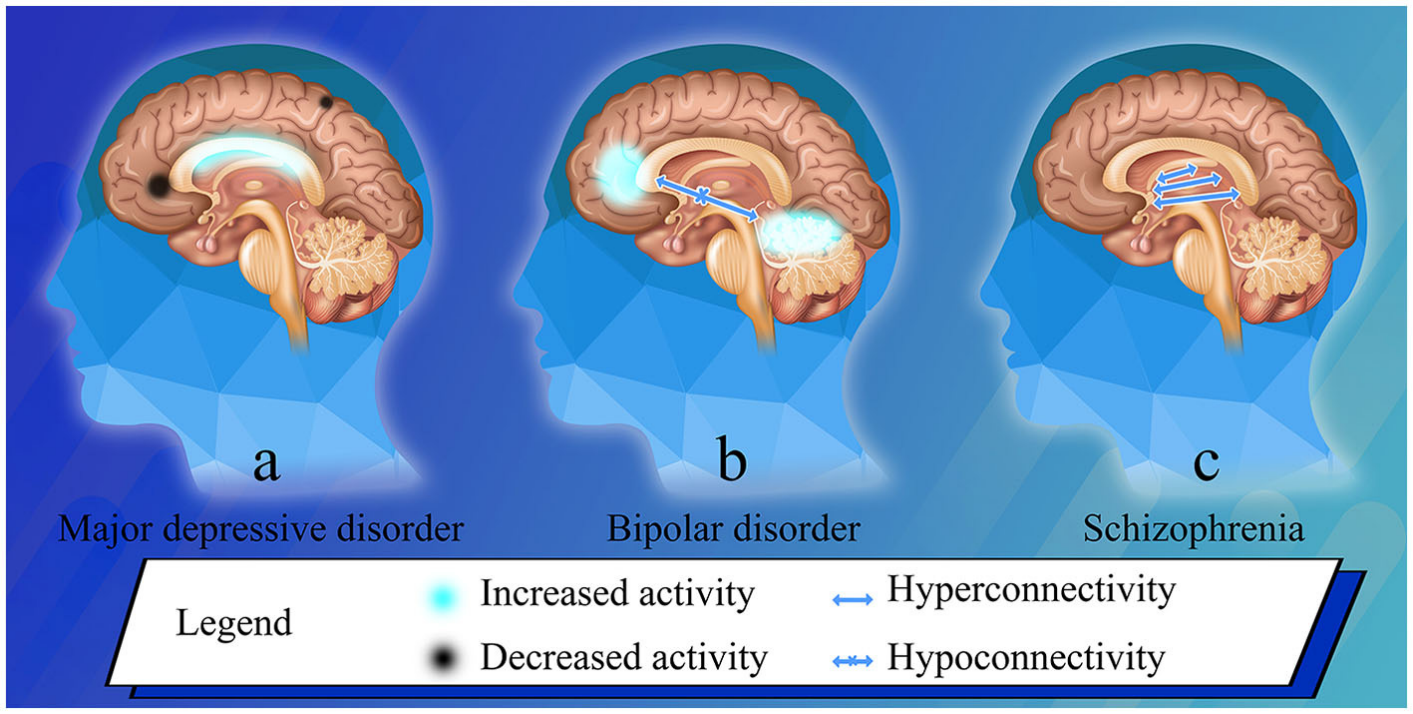
Brain phenotypes in bipolar disorder and schizophrenia, and post-treatment changes in major depressive disorder. **(a)** After ECT in major depressive disorder patients, the low-frequency fluctuation amplitude of the right precentral gyrus significantly reduced; the degree centrality decreased in the left triangle part of the inferior frontal gyrus, and increased in the left hippocampus (1). **(b)** Resting stage functional connectivity between the whole vermis and the ventral prefrontal cortex, and between the anterior vermis and the middle cingulate cortex significantly decreased in bipolar disorder patients (2). **(c)** The regional function increased in the lentiform nucleus, which is related to hyperactivity of dopamine (3).

Neuroimaging and neurostimulation help to translate the neuroscience research from bench to bedside. There are several personal selections of the articles we found particularly interesting and important from a research topic, mapping psychopathology with magnetic resonance imaging (MRI) that contributes to mental health (1–3), involving major psychiatric disorders (Figure 1). Understanding the core MRI-mapped

psychopathology of these disorders, i.e., major depressive disorder, bipolar disorder, and schizophrenia, is critical for diagnosis and treatment. Currently, we still lack the precise treatment strategy to effectively improve the situation.

Converging evidence increasingly suggests that MRI-guided and navigated individualized neurostimulation might be a potential tool in clinical settings (4). Neurostimulation like transcranial direct current stimulation (tDCS), repetitive transcranial magnetic stimulation (rTMS), and electroconvulsive therapy (ECT) employs a brief current or magnetic fields to alter neuronal activity to improve psychiatric symptoms. tDCS, as a relatively new technology, uses different frequency currents to enhance or reduce neuronal activity, while TMS uses high-frequency or low-frequency magnetic fields to enhance or reduce neuronal activity. ECT is a method of brain stimulation with a dose of current below the seizure threshold, which has a wide range of effects on the whole brain, including changes in activity and connectivity in prefrontal cortex (PFC), which are related to treatment and adverse cognitive effects (5). ECT has been proven to be an effective treatment for major depressive disorder and schizophrenia (1), especially for patients with suicidal ideation. Compared with ECT, TMS does not need to cause brain seizures or anesthesia, nor does it cause adverse cognitive effects, but its anti-depressive efficacy is far lower than ECT (5). The choice of neurostimulation method actually depends on what therapeutic purpose can be achieved through the device. Basu et al. (6) combined artificial intelligence with targeted brain stimulation, indicating that precisely targeted neurostimulation can reliably enhance specific human psychological functions related to mental disorders. At the same time, an artificial intelligence algorithm was developed to track the cognitive control ability of patients after stimulation. Based on this, it is possible to explore personalized treatment of mental disorders. MRI can monitor the changes of deep brain structure (7), which is convenient for clinical workers to carry out clinical evaluation. Moreover, due to the development of big data processing, it is possible for the diagnosis and prediction of structural MRI in the field of mental disorders. The updated evidence-based guidelines make recommendations on rTMS for the treatment of depression, post-traumatic stress disorder, schizophrenia, obsessive compulsive disorder, and addiction and craving (8). Major depression, mania, and schizophrenia are principal indications for use of ECT according to the American Psychiatric Association (9). However, core mechanisms of response to rTMS or ECT remain to be identified. A growing number of MRI-based studies elucidating the brain phenotypes can provide insight into the biological mechanisms behind neurostimulation.

## Major depressive disorder

Depression is a common emotional disorder, which is considered to be the functional and structural changes of neural circuits related to emotion, reward, and cognitive processing. The latest findings of structural MRI can use the volume and thickness of cortical gray matter, subcortical volume, and the integrity of white matter bundle to examine the brain structure (or anatomical) changes in neural circuits (10). In the related study of major depressive disorder, it is found that ECT has a certain effect (1). After ECT, amplitude of low frequency fluctuation (ALFF) and fractional ALFF (fALFF) of the right precentral gyrus decreased significantly, and there was a significant correlation between the changes of depressive symptom and ALFF, and between suicidal ideation and fALFF. The degree centrality of the left triangle part of the inferior frontal gyrus and the left hippocampus decreased and increased, respectively. The brain changes detected by MRI may reflect the potential mechanisms behind the efficacy of ECT in adolescent with major depressive disorder. rTMS is also a FDA approved treatment strategy for major depressive disorder. Previous neuroimaging studies have identified rTMS induced changes in brain activity and connectivity in widely distributed brain networks including the default mode network, salience network, reward network, as well as the cognitive control network. More importantly, one of the rTMS responsive regions, the subgenual anterior cingulate cortex has received increasing attention in recent studies. Converging findings have suggested that baseline activity and functional connectivity profiles are associated with treatment efficacy. The results of the meta-analysis on tDCS treatment are inconsistent and further studies are needed to determine the optimal parameters and efficacy of stimulation. rTMS and ECT differ in mechanism, tolerability, and patient acceptability, and they can complement each other rather than competing. There is no maintenance treatment after the completion of standardized neurostimulation treatment, and recurrence is common (11). The duration of neurostimulation treatment for each patient varies from person to person. We need to evaluate the patient's brain structure and resting-state function with MRI (7), whether to implement neurostimulation treatment again to stabilize the patient's symptoms.

## Bipolar disorder

The study by Li et al. (2) is exemplary in exploring the vermal connectivity in bipolar disorder. Although these connections and symptoms were uncorrelated, the study provides some valuable information about dysconnectivity between vermis and ventral PFC. Using the cerebellar vermis as the region of interest, they found that the resting state functional connectivity between the cerebellar vermis and the brain regions involved in emotion regulation changed in patients with bipolar disorder. rTMS achieves therapeutic effect by stimulating these related brain regions which can cause regional changes in neurotransmitter release. Functional MRI can well observe the changes of brain regional circuits, provide a vision for the diagnosis of bipolar disorder by observing biological markers, and help us better understand the biological mechanism of its pathogenesis. Functional MRI has become an effective tool to study the correlation between abnormal functional integrity of human brain at the macroscopic level.

## Schizophrenia

Dr. Ping Li from the Xi'an Mental Health Center begins by calculating the regional brain function of patients with schizophrenia using fALFF (3). She draws attention to the bilateral lentiform nuclei as they relate to cognitive impairments, showing positive correlation with digit span-backward test scores and digit symbol coding scores. The digit span and digit symbol coding tasks could reflect working memory and processing speed, respectively. Cognitive impairment is one of the core characteristics of patients with schizophrenia, which may be due to the dysfunction of dopamine signal transduction circuits in cortex, striatum, and thalamus. Improving the cognitive function of patients with schizophrenia is a key problem in clinical treatment. High frequency TMS of the left PFC can improve negative and cognitive symptoms. Moreover, tDCS can significantly affect neurotransmitters such as dopamine and acetylcholine, weaken dopamine pathway, and effectively reduce the positive symptoms of schizophrenia. The application of MRI before treatment helps to explore the biological subtypes of patients with schizophrenia and predict their response to rTMS according to anatomical structure and functional activation (4). Different frequencies of rTMS treatment can improve the cognitive function of schizophrenia. Under 20 Hz rTMS treatment, the language expression ability and cognitive flexibility are improved. After 10 Hz rTMS treatment, the working memory and long-term memory ability are improved (12). MRI is a potential tool to detect the changes of fALFF in patients with schizophrenia and determine the correlation between abnormal lentiform nucleus function and cognitive impairment in schizophrenia. MRI technology provides a good evaluation index of cognitive function, which will promote the research of neurostimulation to improve the cognitive function of schizophrenia.

## Discussion

Efforts need to be targeted at the macroscopic level to influence cerebral cortex that is committed to changing neuronal activity that underpin the pathogenesis of major psychiatric disorders. Mental disorders are heterogeneous. Symptoms such as “delusions” may present in several different mental disorders, so trying to detect positive symptoms may lead to misdiagnosis. As we avowed earlier (4), MRI shows that different patients present different brain patterns, which can realize accurate planning, positioning, and monitoring of neurostimulation in the future. It is true that it takes a long time for MRI to be applied in clinic, but it is expected to be more accurate, significantly reduce the examination time and improve the patient's tolerance by optimizing the parameters in the pulse sequence to further optimize the MRI method (13). Schizophrenia Imaging Laboratory studies have shown that hundreds of schizophrenia patients can tolerate MRI examination (14). Neurostimulation is still developing, trying to combine with other clinical strategies to improve the symptoms of the disease, and constantly looking for more optimized targets to treat specific symptoms of mental disorders. Segrave et al. (15) combined tDCS with cognitive training can effectively eliminate depressive symptoms and improve cognitive function. The guideline indicates that high-frequency rTMS acting on the primary motor cortex (M1) and the left dorsolateral prefrontal cortex (DLPFC) have clear analgesic and antidepressant effects, respectively (8). The contralateral M1 low frequency rTMS has an obvious effect on the recovery of hand movement after acute stroke. High frequency rTMS acting on the right DLPFC may have effects on negative symptoms of schizophrenia. The strategy of selecting neurostimulation should be combined with the therapeutic purpose to be achieved. In the future, the strategy of combining artificial intelligence algorithm with brain neurostimulation therapy (6), the detailed parameter range composed of various biomarkers should be established on the basis of MRI observation (7), and accurately targeted and appropriate neurostimulation should be used to stimulate the brain related structures according to the abnormal parameters of each person. It has a broad application prospect in individualized treatment (16). Similarly, we are committed to the research of MRI mapping psychopathology, trying to explain the biological basis of various mental disorders by imaging, and can also be used as a mean of testing efficacy. Furthermore, multi-modal MRI, e.g., the structural imaging of gray and white matter (17–19), needs collecting to investigate neuromodulation effects on the brain of patients. Researchers should further study the basis of these mental disorders to provide a new direction for clinical decision-making. We thank them for moving us forward in this direction, as we enthusiastically continue in our pursuit of this critical goal.

## Author contributions

L-BC, BL, and FC conceptualized the manuscript. J-XX and J-JC wrote the first draft of the manuscript.

## Funding

This general commentary was supported by National Natural Science Foundation of China (61976248 and 82271949), Project funded by China Postdoctoral Science Foundation (2020M683739), Health Care Project of Chinese PLA General Hospital (2020ZD05), Basic Research Reinforcement Project (2021-JCJQ-JJ-1079), and the Fourth Military Medical University (2019CYJH).

## Conflict of interest

The authors declare that the research was conducted in the absence of any commercial or financial relationships that could be construed as a potential conflict of interest.

## Publisher's note

All claims expressed in this article are solely those of the authors and do not necessarily represent those of their affiliated organizations, or those of the publisher, the editors and the reviewers. Any product that may be evaluated in this article, or claim that may be made by its manufacturer, is not guaranteed or endorsed by the publisher.
